# Methyl *N*′-[(*E*)-4-hydr­oxy-3-methoxy­benzyl­idene]hydrazinecarboxyl­ate

**DOI:** 10.1107/S1600536808019788

**Published:** 2008-07-05

**Authors:** Xiang-Wei Cheng

**Affiliations:** a Zhejiang Police College Experience Center, Zhejiang Police College, Hangzhou 310053, People’s Republic of China

## Abstract

The mol­ecule of the title compound, C_10_H_12_N_2_O_4_, adopts a *trans* configuration with respect to the C=N double bond. The dihedral angle between the benzene ring and the hydrazinecarboxyl­ate mean plane is 36.54 (6)°. The mol­ecules are linked into a two-dimensional network by inter­molecular O—H⋯O, N—H⋯O and O—H⋯N hydrogen bonds, and by aromatic π–π stacking inter­actions [ring-centroid separation 3.7689 (9) Å].

## Related literature

For a related structure, see: Cheng (2008 ▶).
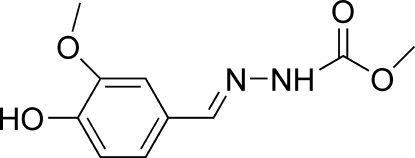


## Experimental

### 

#### Crystal data

C_10_H_12_N_2_O_4_
*M* *_r_* = 224.22
Monoclinic, 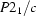

*a* = 9.4718 (10) Å
*b* = 11.0983 (11) Å
*c* = 10.3220 (11) Å
β = 98.272 (4)°
*V* = 1073.77 (19) Å^3^
*Z* = 4
Mo *K*α radiation
μ = 0.11 mm^−1^
*T* = 123 (2) K
0.29 × 0.27 × 0.26 mm

#### Data collection

Bruker SMART CCD diffractometer
Absorption correction: multi-scan (*SADABS*; Bruker, 2002 ▶) *T*_min_ = 0.965, *T*_max_ = 0.968
11214 measured reflections
1887 independent reflections
1590 reflections with *I* > 2σ(*I*)
*R*_int_ = 0.029

#### Refinement

*R*[*F* ^2^ > 2σ(*F* ^2^)] = 0.035
*wR*(*F* ^2^) = 0.113
*S* = 1.02
1887 reflections
146 parameters
H-atom parameters constrained
Δρ_max_ = 0.19 e Å^−3^
Δρ_min_ = −0.14 e Å^−3^

### 

Data collection: *SMART* (Bruker, 2002 ▶); cell refinement: *SAINT* (Bruker, 2002 ▶); data reduction: *SAINT*; program(s) used to solve structure: *SHELXS97* (Sheldrick, 2008 ▶); program(s) used to refine structure: *SHELXL97* (Sheldrick, 2008 ▶); molecular graphics: *SHELXTL* (Sheldrick, 2008 ▶); software used to prepare material for publication: *SHELXTL*.

## Supplementary Material

Crystal structure: contains datablocks I, global. DOI: 10.1107/S1600536808019788/hb2754sup1.cif

Structure factors: contains datablocks I. DOI: 10.1107/S1600536808019788/hb2754Isup2.hkl

Additional supplementary materials:  crystallographic information; 3D view; checkCIF report

## Figures and Tables

**Table 1:** Hydrogen-bond geometry (Å, °)

*D*—H⋯*A*	*D*—H	H⋯*A*	*D*⋯*A*	*D*—H⋯*A*
O1—H1⋯O2	0.84	2.21	2.6695 (15)	114
O1—H1⋯O3^i^	0.84	2.34	3.0286 (16)	139
O1—H1⋯N1^i^	0.84	2.57	3.2640 (17)	140
N2—H2*B*⋯O3^ii^	0.88	2.19	3.0124 (16)	155

Symmetry codes: (i) 
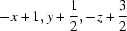
; (ii) 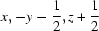.

**Fig. 1.**
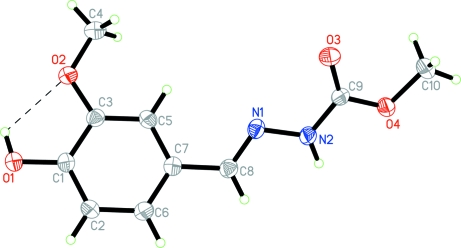
The molecular structure of (I), showing 30% probability displacement ellipsoids for the non-hydrogen atoms.

## supplementary crystallographic information

### Comment

As part of our ongoig studies of benzaldehydehydrazone derivatives (Cheng, 2008), we now report the synthesis and structure of the title compound, (I).

The title molecule (Fig. 1) adopts a *trans* configuration with respect to the C═N bond.  The dihedral angle between the benzene ring and the C9/C10//N1/N2/O3/O4 plane is 36.54 (6)°. Otherwise, the bond lengths and angles for (I) agree with those observed for (*E*)-methyl*N*'-(4-hydroxybenzylidene)hydrazinecarboxylate (Cheng, 2008).

In the crystal, the molecules are linked into a two-dimensional network by intramolecular O—H···O and intermolecular O—H···O, N—H···O, O—H···N hydrogen bonds (Table 1). Additionally, neighbouring aromatic rings interact by π–π stacking [centroid separation = 3.7689 (9) Å].

### Experimental

4-Hydroxy-3-methoxybenzaldehyde (1.52 g, 0.01 mol) and methyl hydrazinecarboxylate (0.9 g, 0.01 mol) were dissolved in stirred methanol (15 ml) and left for 2.5 h at room temperature. The resulting solid was filtered off and recrystallized from ethanol to give the title compound in 90% yield. Colourless blocks of (I) were obtained by slow evaporation of a ethanol solution at room temperature (m.p. 480–482 K).

### Refinement

The H atoms were placed geometrically (O—H = 0.84 Å, N—H = 0.88 Å and C—H = 0.95 or 0.98 Å) and refined as riding, with *U*_iso_(H) = 1.2 or 1.5*U*_eq_(carrier).

### Crystal data

**Table:**

C_10_H_12_N_2_O_4_	*F*_000_ = 472
*M**_r_* = 224.22	*D*_x_ = 1.387 Mg m^−^^3^
Monoclinic, *P*2_1_/*c*	Mo *K*α radiation λ = 0.71073 Å
Hall symbol: -P 2ybc	Cell parameters from 1887 reflections
*a* = 9.4718 (10) Å	θ = 2.2–25.0º
*b* = 11.0983 (11) Å	µ = 0.11 mm^−^^1^
*c* = 10.3220 (11) Å	*T* = 123 (2) K
β = 98.272 (4)º	Block, colourless
*V* = 1073.77 (19) Å^3^	0.29 × 0.27 × 0.26 mm
*Z* = 4	

### Data collection

**Table:**

Bruker SMART CCD diffractometer	1887 independent reflections
Radiation source: fine-focus sealed tube	1590 reflections with *I* > 2σ(*I*)
Monochromator: graphite	*R*_int_ = 0.029
*T* = 123(2) K	θ_max_ = 25.0º
ω scans	θ_min_ = 2.2º
Absorption correction: multi-scan(SADABS; Bruker, 2002)	*h* = −11→10
*T*_min_ = 0.965, *T*_max_ = 0.968	*k* = −13→13
11214 measured reflections	*l* = −11→12

### Refinement

**Table:**

Refinement on *F*^2^	Hydrogen site location: inferred from neighbouring sites
Least-squares matrix: full	H-atom parameters constrained
*R*[*F*^2^ > 2σ(*F*^2^)] = 0.035	*w* = 1/[σ^2^(*F*_o_^2^) + (0.0687*P*)^2^ + 0.1817*P*] where *P* = (*F*_o_^2^ + 2*F*_c_^2^)/3
*wR*(*F*^2^) = 0.113	(Δ/σ)_max_ = 0.010
*S* = 1.02	Δρ_max_ = 0.19 e Å^−^^3^
1887 reflections	Δρ_min_ = −0.13 e Å^−^^3^
146 parameters	Extinction correction: SHELXL97 (Sheldrick, 2008), Fc^*^=kFc[1+0.001xFc^2^λ^3^/sin(2θ)]^-1/4^
Primary atom site location: structure-invariant direct methods	Extinction coefficient: 0.036 (5)
Secondary atom site location: difference Fourier map	

### Special details

**Table:**

Geometry. All e.s.d.'s (except the e.s.d. in the dihedral angle between two l.s. planes) are estimated using the full covariance matrix. The cell e.s.d.'s are taken into account individually in the estimation of e.s.d.'s in distances, angles and torsion angles; correlations between e.s.d.'s in cell parameters are only used when they are defined by crystal symmetry. An approximate (isotropic) treatment of cell e.s.d.'s is used for estimating e.s.d.'s involving l.s. planes.
Refinement. Refinement of *F*^2^ against ALL reflections. The weighted *R*-factor *wR* and goodness of fit *S* are based on *F*^2^, conventional *R*-factors *R* are based on *F*, with *F* set to zero for negative *F*^2^. The threshold expression of *F*^2^ > σ(*F*^2^) is used only for calculating *R*-factors(gt) *etc*. and is not relevant to the choice of reflections for refinement. *R*-factors based on *F*^2^ are statistically about twice as large as those based on *F*, and *R*- factors based on ALL data will be even larger.

### Fractional atomic coordinates and isotropic or equivalent isotropic displacement parameters (Å^2^)

**Table:**

	*x*	*y*	*z*	*U*_iso_*/*U*_eq_	
C1	0.46841 (16)	0.22471 (12)	0.90488 (15)	0.0408 (4)	
C2	0.57541 (17)	0.23855 (13)	1.00956 (16)	0.0453 (4)	
H2A	0.5760	0.3073	1.0645	0.054*	
C3	0.46883 (16)	0.12325 (13)	0.82397 (14)	0.0405 (4)	
C4	0.3402 (2)	0.01177 (17)	0.64655 (17)	0.0596 (5)	
H4A	0.2550	0.0191	0.5809	0.089*	
H4B	0.3309	−0.0589	0.7016	0.089*	
H4C	0.4246	0.0026	0.6025	0.089*	
C5	0.57560 (15)	0.03861 (13)	0.84778 (14)	0.0406 (4)	
H5	0.5761	−0.0291	0.7916	0.049*	
C6	0.68260 (16)	0.15212 (14)	1.03504 (15)	0.0439 (4)	
H6	0.7554	0.1618	1.1079	0.053*	
C7	0.68361 (15)	0.05223 (13)	0.95482 (14)	0.0390 (4)	
C8	0.79364 (16)	−0.04036 (13)	0.98484 (15)	0.0425 (4)	
H8	0.8497	−0.0414	1.0689	0.051*	
C9	0.96010 (15)	−0.28026 (12)	0.85416 (14)	0.0381 (4)	
C10	1.0957 (2)	−0.45396 (18)	0.82782 (19)	0.0681 (6)	
H10A	1.1524	−0.5152	0.8804	0.102*	
H10B	1.1562	−0.4113	0.7734	0.102*	
H10C	1.0166	−0.4929	0.7715	0.102*	
N1	0.81595 (13)	−0.11952 (11)	0.90087 (12)	0.0409 (3)	
N2	0.91622 (13)	−0.20662 (11)	0.94426 (12)	0.0434 (3)	
H2B	0.9502	−0.2137	1.0279	0.052*	
O1	0.36374 (12)	0.30938 (9)	0.88270 (11)	0.0543 (3)	
H1	0.3060	0.2900	0.8164	0.081*	
O2	0.35509 (13)	0.11728 (10)	0.72612 (11)	0.0571 (4)	
O3	0.93369 (12)	−0.26695 (10)	0.73659 (10)	0.0508 (3)	
O4	1.03976 (12)	−0.36926 (10)	0.91342 (10)	0.0520 (3)	

### Atomic displacement parameters (Å^2^)

**Table:**

	*U*^11^	*U*^22^	*U*^33^	*U*^12^	*U*^13^	*U*^23^
C1	0.0423 (8)	0.0368 (7)	0.0437 (9)	0.0025 (6)	0.0071 (6)	0.0028 (6)
C2	0.0511 (9)	0.0373 (8)	0.0469 (9)	0.0004 (6)	0.0045 (7)	−0.0050 (6)
C3	0.0410 (8)	0.0445 (8)	0.0360 (8)	0.0010 (6)	0.0055 (6)	0.0012 (6)
C4	0.0648 (11)	0.0618 (10)	0.0485 (10)	0.0017 (9)	−0.0038 (8)	−0.0113 (8)
C5	0.0432 (8)	0.0401 (8)	0.0398 (8)	0.0016 (6)	0.0101 (6)	−0.0025 (6)
C6	0.0431 (8)	0.0458 (8)	0.0415 (9)	−0.0022 (7)	0.0018 (6)	0.0014 (6)
C7	0.0387 (8)	0.0403 (7)	0.0390 (8)	−0.0002 (6)	0.0095 (6)	0.0040 (6)
C8	0.0431 (8)	0.0455 (8)	0.0387 (8)	0.0030 (6)	0.0056 (6)	0.0025 (6)
C9	0.0352 (7)	0.0434 (8)	0.0349 (8)	−0.0014 (6)	0.0029 (6)	0.0006 (6)
C10	0.0767 (13)	0.0687 (12)	0.0567 (11)	0.0307 (10)	0.0019 (9)	−0.0149 (9)
N1	0.0393 (7)	0.0442 (7)	0.0393 (7)	0.0059 (5)	0.0064 (5)	0.0070 (5)
N2	0.0465 (7)	0.0500 (7)	0.0328 (7)	0.0132 (6)	0.0033 (5)	0.0023 (5)
O1	0.0565 (7)	0.0465 (6)	0.0559 (7)	0.0137 (5)	−0.0053 (5)	−0.0084 (5)
O2	0.0551 (7)	0.0610 (7)	0.0501 (7)	0.0157 (5)	−0.0093 (5)	−0.0147 (5)
O3	0.0589 (7)	0.0593 (7)	0.0336 (7)	0.0073 (5)	0.0046 (5)	0.0001 (5)
O4	0.0612 (7)	0.0534 (7)	0.0402 (6)	0.0199 (5)	0.0026 (5)	−0.0030 (5)

### Geometric parameters (Å, °)

**Table:**

C1—O1	1.3613 (17)	C6—H6	0.9500
C1—C2	1.380 (2)	C7—C8	1.465 (2)
C1—C3	1.402 (2)	C8—N1	1.2729 (19)
C2—C6	1.394 (2)	C8—H8	0.9500
C2—H2A	0.9500	C9—O3	1.2123 (18)
C3—O2	1.3682 (18)	C9—O4	1.3366 (17)
C3—C5	1.376 (2)	C9—N2	1.3479 (19)
C4—O2	1.4256 (19)	C10—O4	1.4421 (19)
C4—H4A	0.9800	C10—H10A	0.9800
C4—H4B	0.9800	C10—H10B	0.9800
C4—H4C	0.9800	C10—H10C	0.9800
C5—C7	1.402 (2)	N1—N2	1.3837 (16)
C5—H5	0.9500	N2—H2B	0.8800
C6—C7	1.385 (2)	O1—H1	0.8400
			
O1—C1—C2	119.35 (13)	C6—C7—C8	120.08 (13)
O1—C1—C3	121.23 (13)	C5—C7—C8	120.50 (13)
C2—C1—C3	119.42 (13)	N1—C8—C7	121.53 (13)
C1—C2—C6	120.28 (14)	N1—C8—H8	119.2
C1—C2—H2A	119.9	C7—C8—H8	119.2
C6—C2—H2A	119.9	O3—C9—O4	124.73 (13)
O2—C3—C5	125.38 (13)	O3—C9—N2	125.25 (13)
O2—C3—C1	114.15 (13)	O4—C9—N2	110.01 (12)
C5—C3—C1	120.45 (13)	O4—C10—H10A	109.5
O2—C4—H4A	109.5	O4—C10—H10B	109.5
O2—C4—H4B	109.5	H10A—C10—H10B	109.5
H4A—C4—H4B	109.5	O4—C10—H10C	109.5
O2—C4—H4C	109.5	H10A—C10—H10C	109.5
H4A—C4—H4C	109.5	H10B—C10—H10C	109.5
H4B—C4—H4C	109.5	C8—N1—N2	115.80 (12)
C3—C5—C7	120.09 (13)	C9—N2—N1	117.75 (12)
C3—C5—H5	120.0	C9—N2—H2B	121.1
C7—C5—H5	120.0	N1—N2—H2B	121.1
C7—C6—C2	120.37 (14)	C1—O1—H1	109.5
C7—C6—H6	119.8	C3—O2—C4	117.85 (12)
C2—C6—H6	119.8	C9—O4—C10	115.75 (12)
C6—C7—C5	119.38 (13)		
			
O1—C1—C2—C6	179.17 (14)	C3—C5—C7—C8	176.91 (13)
C3—C1—C2—C6	−0.3 (2)	C6—C7—C8—N1	−165.42 (14)
O1—C1—C3—O2	−1.5 (2)	C5—C7—C8—N1	16.9 (2)
C2—C1—C3—O2	177.96 (14)	C7—C8—N1—N2	−175.85 (12)
O1—C1—C3—C5	179.93 (14)	O3—C9—N2—N1	10.3 (2)
C2—C1—C3—C5	−0.6 (2)	O4—C9—N2—N1	−170.91 (11)
O2—C3—C5—C7	−177.24 (13)	C8—N1—N2—C9	−169.52 (13)
C1—C3—C5—C7	1.1 (2)	C5—C3—O2—C4	4.0 (2)
C1—C2—C6—C7	0.7 (2)	C1—C3—O2—C4	−174.49 (14)
C2—C6—C7—C5	−0.1 (2)	O3—C9—O4—C10	−0.3 (2)
C2—C6—C7—C8	−177.83 (13)	N2—C9—O4—C10	−179.09 (14)
C3—C5—C7—C6	−0.8 (2)		

### Hydrogen-bond geometry (Å, °)

**Table:**

*D*—H···*A*	*D*—H	H···*A*	*D*···*A*	*D*—H···*A*
O1—H1···O2	0.84	2.21	2.6695 (15)	114
O1—H1···O3^i^	0.84	2.34	3.0286 (16)	139
O1—H1···N1^i^	0.84	2.57	3.2640 (17)	140
N2—H2B···O3^ii^	0.88	2.19	3.0124 (16)	155

Symmetry codes: (i) −*x*+1, *y*+1/2, −*z*+3/2; (ii) *x*, −*y*−1/2, *z*+1/2.

## References

[bb1] Bruker (2002). *SADABS*, *SMART* and *SAINT* Bruker AXS Inc., Madison, Wisconsin, USA.

[bb2] Cheng, X.-W. (2008). *Acta Cryst.* E**64**, o1302. 10.1107/S1600536808018096 PMC2961885 21202931

[bb3] Sheldrick, G. M. (2008). *Acta Cryst.* A**64**, 112–122. 10.1107/S0108767307043930 18156677

